# DRD4 gene polymorphism and impulse control disorder induced by dopamine agonists in Parkinson's disease

**DOI:** 10.1002/acn3.52111

**Published:** 2024-07-01

**Authors:** Viviana Torres, Jesica Pérez‐Montesino, Rubén Fernández‐Santiago, Manel Fernández, Ana Camara, Yaroslau Compta, María‐José Martí, Àlex Guerra Beltran, José Rios, Francesc Valldeoriola, Mario Ezquerra

**Affiliations:** ^1^ Parkinson's Disease and Movement Disorders Unit, Neurology Service Institut de Neurociencies UBNeuro, Hospital Clínic Universitari de Barcelona Barcelona Catalonia Spain; ^2^ Lab of Parkinson's disease and other Neurodegenerative Movement Disorders: Clinical and Experimental Research Institut d'Investigacions Biomèdiques August Pi I Sunyer (IDIBAPS) Barcelona 08036 Catalonia Spain; ^3^ Department of Clinical Pharmacology, Hospital Clinic, and Medical Statistics Core Facility Institutd'InvestigacionsBiomèdiques August Pi ISunyer (IDIBAPS) Barcelona Catalonia Spain; ^4^ Biostatistics Unit, School of Medicine UniversitatAutònoma de Barcelona Barcelona Catalonia Spain

## Abstract

Impulse control disorders and their consequences display variability among individuals, indicating potential involvement of environmental and genetic factors. In this retrospective study, we analyzed a cohort of Parkinson's disease patients treated with dopamine agonists and investigated the influence of the dopamine D4 receptor gene polymorphism, DRD4 7R+, which is linked to psychiatric disorders, impulsive traits, and addictive behaviors. We found that DRD4 7R+ is a significant genetic risk factor associated with the severity of ICD.

## Introduction

Parkinson's disease (PD) is the most frequent neurodegenerative parkinsonism. To delay the onset of early‐onset motor complications caused using L‐dopa,^1^ dopamine agonists (DAs) have been frequently prescribed, showing less frequent development of wearing off, dyskinesias, or on–off motor fluctuations. Pramipexole, highlighted as an example, lowers dyskinesia risk (28% vs. levodopa's 51%)^2^; other agonists show similar benefits. Several large, randomized trials have demonstrated the efficacy of DAs as monotherapy in the early stages of PD and adjunct therapy with L‐dopa in advanced stages. However, in turn, DA treatment has been associated with impulse control disorders (ICDs) incidence in a subset of patients.^3^

PD patients and their families can experience severe consequences due to ICDs, including economic, legal, and familial effects. Therefore, health care providers must identify ICD risk factors before administering DA to guide preventive strategies. Some studies have shown genetic associations with ICD susceptibility in PD patients, and several genes related to dopaminergic, serotoninergic, opioid, and norepinephrine systems have been linked as leading candidates.^4^, ^5^, ^6^ Among them, the dopamine receptor gene 4 (DRD4) appears to be involved in human behavior. This gene is characterized by a 48 base‐pair variable number of tandem repeats (48‐bp VNTR) in the coding region, varying from 2 to 11 repetitions across individuals.^7^ Subjects with seven or longer repeats number (DRD4 7+) showed an increased risk for neuropsychiatric diseases, compulsive, addictive, delinquent behaviors, and personality traits such as novelty‐seeking, financial risk, binge eating, and increased gambling propensity after dopaminergic stimulation.^8^, ^9^, ^10^

The present study aimed at analyzing the association between the DRD4 VNTR and the appearance of ICD in DA‐treated PD patients to provide additional genetic predictive factors for personalizing therapeutic approaches avoiding the use of dopamine agonists in ICD at‐risk subpopulations.

## Methods

### Study design and clinical data

We conducted an observational retrospective study at Hospital Clinic Universitari de Barcelona, analyzing a well‐defined cohort of patients whose biological samples were available in the repository. This cohort comprised individuals who had been evaluated in the Parkinson's and Movement Disorders Unit from 2004 to the present. The clinical data, available in electronic format, was reviewed by a neurologist specialized in movement disorders, and eventually a neuropsychiatrist. A complete explanation of patients' inclusion and exclusion criteria and experimental details is provided (Fig. S1).

Our study included 241 patients, mostly of European origin (94.6%), with a clinical diagnosis of PD, treated with DA, with or without ICD up to the assessment. Table 1 shows the clinical characteristics of the cohort and DRD4 genotypes stratified by ICD status. To identify a potential history of ICD, we used the Questionnaire for Impulsive‐Compulsive Disorders in Parkinson's disease—current Short (QUIP‐CS) for compulsive gambling, sex, buying, eating, and other behaviors. The QUIP‐CS questionnaire is a standard part of the patient history‐taking process during consultations with our medical team. The cohort was classified into three categories: absence of impulse control behaviors (non‐ICBs), presence of impulse control behaviors (ICBs), and impulse control disorders (ICDs). The ICBs category included patients who did not meet the severity criteria. Meanwhile, ICDs were defined as behaviors that resulted in significant or severe consequences like family or work dysfunction, compromising the quality of life, including hypersexuality affecting couples and their environment, prostitution, weight gain greater than 5 kg, or financial insolvency. Therefore, we will refer to patients with any type of impulsive‐compulsive behavior or disorder as ICB‐ICD.

**Table 1:** Demographic, clinical, and genetic characteristics of our cohort (*N* = 241) grouped by ICB‐ICD and ICD development.

	Grouped by ICB‐ICD		Grouped by ICD	
	Non‐ICB *N* = 137 (56.85%)	ICB‐ICD *N* = 104 (43.15%)	Non‐ICD *N* = 182 (75.52%)	ICD *N* = 59 (24.48%)
	Frequency (%)	Frequency (%)	Frequency (%)	Frequency (%)
*Characteristic*				
Sex (male)	79 (57.66%)^d^	75 (72.12%)^d^	108 (59.34%)^d^	46 (77.97%)^d^
Modified H&Y stage	2 (1–2)^a^	2 (1–2)^a^	2 (1–2)^a^	2 (1–2)^a^
Age of PD onset (years)	53.6 (10.4)^b^	49.2 (10.7)^b^	53.1 (10.3)^b^	47.4 (11.1)^b^
DA therapy total duration (years)	7.6 (4.9)^b^	3.7 (3.2)^b^	6.6 (4.8)^b^	3.7 (3.5)^b^
Depression or anxiety	7 (5.11%)^d^	9 (8.65%)^d^	10 (5.49%)^d^	6 (10.17%)^d^
Clinical history RBD+	47 (34.31%)^d^	54 (51.92%)^d^	70 (38.46%)^d^	31 (52.54%)^d^
Dyskinesias	28 (11.60%)^d^	35 (14.50%)^d^	38 (20.88%)^d^	25 (42.37%)^d^
LEDD (mg)	952.4 (383.2)^b^	927.5 (344.6)^b^	934.6 (370.1)^b^	963.2 (357.6)^b^
LED DA (mg)	217.5 (108.8)^b^	241.3 (105.1)^b^	219.9 (106.9)^b^	252.1 (107.7)^b^
DRD4 7‐	108 (78.83%)^d^	74 (71.15%)^d^	145 (79.67%)^d^	37 (62.71%)^d^
DRD4 7+	29 (21.17%)^d^	30 (28.85%)^d^	37 (20.33%)^d^	22 (37.29%)^d^
Time from DA initiation to ICB‐ICDs (years)	N/A	2.9 (2.5)^b^	3.1 (2.5)^b^, ^c^	2.7 (2.5)^b^
Time from PD onset to ICB‐ICDs (years)	N/A	6.6 (5.2)^b^	7.1 (5.7)^b^, ^c^	6.3 (4.8)^b^
Disease duration (years)	17.3 (7.86)^b^	18.1 (9.80)^b^	17.4 (8.08)^b^	18.4 (10.4)^b^

^a^ Median (IQR).

^b^ Mean (SD).

^c^ Calculated only with ICBs patients (45/182).

^d^ Percentages % are calculated from the *N* of each subgroup.

We collected sociodemographic and clinical data from the charts for each patient, including sex, age, marital status, and psychiatric comorbidity. Psychiatric comorbidity was assessed based on diagnoses recorded in the patients' medical records, established by specialists in mental health. For PD‐related variables, we collected data on age at PD onset, Hoehn &Yahr (H&Y) at the starting time of therapy institution, time from PD onset to ICB‐ICDs, presence of moderate–severe dyskinesias, and clinical history of eye movement (REM) sleep behavior disorder (RBD). The presence or absence of RBD was determined based on the RBD‐Single‐Question Screen as part of our routine patient consultations.

Treatment history was collected from medical charts and prescriptions, describing the implicated DA, the duration of treatment, and time from DA treatment onset to ICB‐ICDs if present, in which case we calculated both maximum L‐dopa equivalent daily doses (LEDDs) and L‐dopa equivalent dose of dopamine agonist (LED DA).

We classified the type of ICB‐ICDs as binge eating, money‐related disorders (gambling or buying), hypersexuality, and others ICB‐ICDs such as impulsivity and punding. The local ethics committee of Hospital Clínic Universitari de Barcelona approved the study.

### Genetic analysis

DNA was extracted from peripheral blood following the Wizard® Genomic DNA Purification Kit Protocol (Promega Corp., WI, USA). Dopamine D4 receptor gene polymorphism (DRD4 VNTR) was amplified and genotyped for each DNA sample as previously described,^6^ using the primers 5′‐GCGACTACGTGG TCTACT CG‐3′ (forward) and 5′‐AGGACCCTCATGGCC TTG‐3′ (reverse) in a VeritiTM 96‐well Thermal Cycle (Applied Biosystems #4375786). Full genetic analysis methodology is provided in Supplementary Material.

### Statistical analysis

We stratified patients according to three groups: non‐ICBs, ICBs, or ICDs. As no significant differences in any variable were found between non‐ICBs and ICBs groups, we combined them for subsequent analysis (non‐ICD) comparing with ICD (Table S1), allowing for exploring the genotype effect on impulsive‐compulsive severity (non‐ICD versus ICD) besides on its presence. Full statistical analysis methods can be found in the Supplementary Material.

## Results

### Demographic analysis

Out of 241 DA‐treated PD patients, 104 patients (43.15%) developed ICB‐ICDs, with 29 women (12.03%) and 75 men (31.12%) affected. Of all ICB‐ICDs patients, about 45 (18.67%) developed ICBs, while 59 (24.48%) developed ICDs. The mean age at PD onset was 49.2 years (±10.7 years) for patients with ICB‐ICDs and 53.6 years (±10.4 years) for those without. Patients with ICD were significantly younger at disease diagnosis (*p* < 0.001). Depression or anxiety as psychiatric comorbidity was reported in 16 individuals, accounting for 6.64% of the cohort. There was no significant difference in LEDD mean between individuals with ICBs or non‐ICBs and those with ICD (*p* = 0.701). The most used DA drug in both ICD and non‐ICD patients was pramipexole (61.54% and 54.74%, respectively), followed by rotigotine (24.04% and 24.09%, respectively) and others DA including apomorphine, cabergoline and andropirinole (14.42% and 21.17%, respectively). Among the different ICD types, hypersexuality and money‐related disorders were the most frequent ones, each occurring in (*n* = 41/104) 39.42% of ICB‐ICD cases. Money‐related disorders were more common in women (*n* = 13/29; 44.83%), while hypersexuality was more common in men (*n* = 37/75; 49.33%). Almost one in four patients (*n* = 23/104; 22.12%) developed multiple types of ICDs.

Allele and genotype frequency distribution for the DRD4 polymorphism of DRD4 are described in Table S2.

### Univariate cox analysis

Univariate Cox regression showed a significant association of DRD4 7+ with ICD (HR 2.00, 95% CI 1.18–3.39, *p* = 0.010) alongside younger age at PD onset (HR 0.96, 95% CI 0.94–0.98, *p* < 0.001), male sex (HR 2.38, 95% CI 1.18–4.41, *p* = 0.006), psychiatric diseases (HR 2.60, 95% CI 1.11–6.09, *p* = 0.028), and moderate and severe dyskinesia (HR 2.27, 95% CI 1.35–3.80, *p* = 0.002) (Table S3). There were no significant differences in the amount of LEDD or LED DA for ICD development. While RBD did not attain statistical significance (*p* = 0.092), the subtle trend observed hints a potential association, suggesting a marginal increased risk of ICD outcome for patients with RBD. This finding can be further explored in future investigations with larger sample sizes and refined RBD testing methodologies.

### Multivariable cox analysis

**Figure 1:**
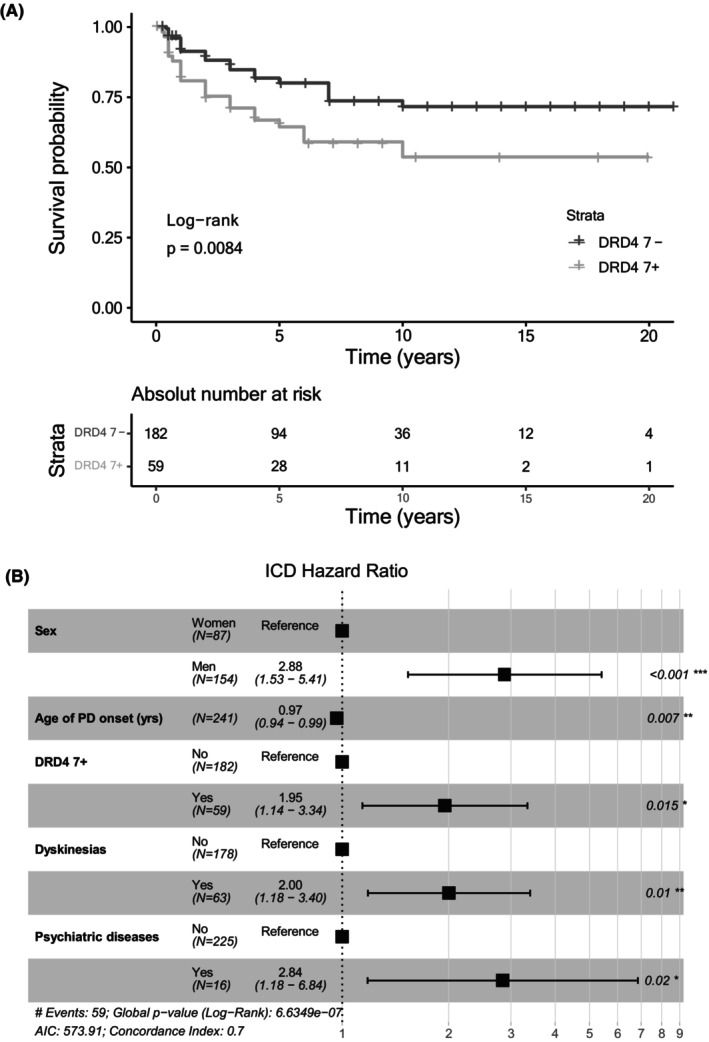
Survival analysis of ICD. (A) Kaplan–Meier survival plot showing the delay of ICD appearance in patients without DRD4 7− comparing with 7+ genotype, and table showing the absolute number at risk in each time interval. (B) Forest plot showing multivariate Cox regression analysis of ICD survival for all significant risk factors and their respective hazard ratios. ***p < 0.001, **p < 0.01, *p < 0.05.

Multivariable Cox regression revealed that DRD4 7+ was statistically significant associated with risk of ICD development (HR 1.95, 95% CI 1.14–3.34, *p* = 0.015) along with moderate and severe dyskinesias (HR 2.00, 95% CI 1.18–3.39, *p* = 0.010), male sex (HR 2.88, 95% CI 1.53–5.41, *p* < 0.001), and psychiatric diseases (HR 2.84, 95% CI 1.18–6.84, *p* = 0.020) (Fig. 1). Furthermore, the age of PD onset was negatively correlated with the risk of ICD, with each year of the age of onset decreasing the risk by 3% (HR 0.97, 95% CI 0.94–0.99, *p* = 0.007).

### Logistic regression model

**Figure 2:**
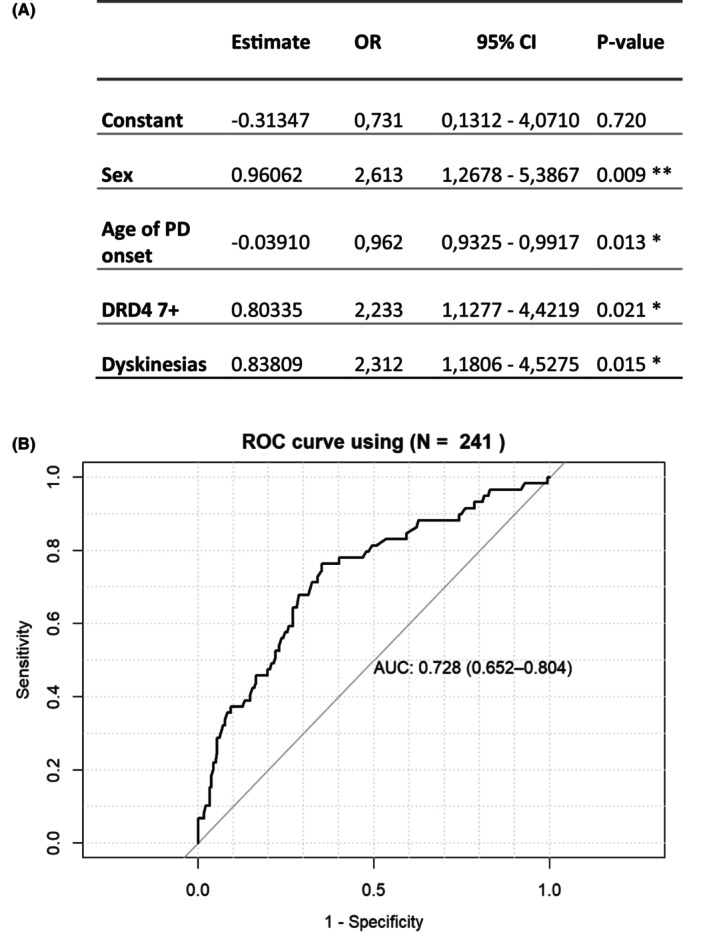
(A) Summary of the logistic regression model designed to assess the development of ICD. The table displays estimates, odds ratios (OR), and 95% confidence intervals (CI) for each variable within the model. Sex is represented as male versus female, with male being associated with a higher risk of ICD (positive estimate). Asterisks denote statistical significance levels: *p < 0.05, **p < 0.01. (B) Area under the curve (AUC) analysis and 95% CI on a receiver operating characteristic (ROC) curve representing the discriminative ability of sex, age PD onset, DRD4 polymorphism, and dyskinesia in PD patients on DA treatment when comparing ICD versus non‐ICD.

We included in the multivariable logistic regression equation sex, age of PD onset, DRD4 7+, and dyskinesias as covariates, and the presence of ICD versus non‐ICBs and ICBs as the dependent variable. The significant variables associated with ICD development were sex (OR 2.61; CI 1.26–5.39, *p* = 0.009), age at PD onset (OR 0.96; CI 0.93–0.99, *p* = 0.013), DRD4 7+ polymorphism (OR 2.23; CI 1.13–4.42, *p* = 0.021), and presence of dyskinesias (OR 2.31; CI 1.18–4.53, *p* = 0.015). Finally, we assessed the discriminative capacity of these risk factors to identify ICD development, observing an AUC of 0.73 (95%CI, 0.65–0.8) (Fig. 2).

## Discussion

Our study suggests that the DRD4 7+ polymorphism is associated with a specific ICD phenotype characterized by the presence of severe social consequences in Parkinson's patients treated with DA. The positive association was independent of sex, age at disease onset, and either DAs or equivalent daily levodopa dose. Although previous studies have explored the possible link between DRD4 gene variants and the pathogenesis and etiology of PD,^11^, ^12^ this is the first study researching the possible effect of DRD4 on drug‐induced ICD in PD patients.

We have replicated previously identified risk factors for ICB‐ICDs in PD, including early disease onset, male sex, psychiatric comorbidities, moderate and severe dyskinesia,^13^ and symptoms of RBD.^14^ These results are consistent with both Cox regression analysis and the logistic regression model. The lack of significance of related psychiatric diseases variable in the logistic regression model but significant in the Cox analysis may suggest a time‐dependent relationship between this variable and ICD survival or a potential sample limitation (6.64% of the entire cohort). After 5 years of DA treatment, 40.4% of patients in our study developed ICB‐ICDs (*n* = 97/241), with an 84.7% of them being severe cases (ICDs). Notably, none of the patients developed ICDs after 10 years of DA treatment. These findings are consistent with prior research reporting a 5‐year cumulative incidence of ICB‐ICDs of 45% and indicating that ICB‐ICDs are more likely to occur in the early stages of PD when the use of DA is more frequent, with their prevalence stabilizing over time.^15^ Contrary to earlier research that suggested a relationship between DA dosage or LEDD and the development of ICB‐ICDs,^3^ our results suggest that exposure to any DA dose could be enough to cause ICD, at least in susceptible individuals. Our results suggest that exposure to any DA dose could be enough to cause ICD, at least in susceptible individuals. In agreement with this idea, *recent research indicates that the use of any dose of specific DA drugs such as pramipexol may in itself confer high ICD risk*.^16^

The *DRD4* gene encodes the D4 subtype of the dopamine receptor, which is predominantly expressed in the deep layer neurons in the prefrontal cortex (PFC). The receptor isoforms encoded by this gene exhibit different abilities to modulate the inhibitory dopaminergic neurotransmission in the frontal corticostriatal circuit.^17^ It has been suggested that the DRD4 7+ variant exerts its effects by forming heteromers with DRD2, resulting in increased potency of dopamine‐mediated inhibition of glutamate. In rodents, this leads to overactivity in the mesocorticolimbic pathway and a greater tendency toward impulsive behaviors.^18^

Our research has various strengths, including the use of a well, clinically characterized and homogeneous cohort of subjects. However, we acknowledge some limitations. The study was retrospective with a high number of patients excluded (*n* = 657). The assessment of the presence of ICD social consequences was conducted through clinical interviews as part of routine practice, but it lacked a standardized approach. Nevertheless, it is worth mentioning that the definition of ICD severity employed aligns with the recent classification criteria proposed by the International Expert Consensus on ICBD treatment strategies.^19^ This classification system categorizes severity based on the impact on the biopsychosocial functioning of the patient or their significant others, providing a clinically relevant framework for assessing the severity of ICDs. It is also important to consider that the severity of ICD may vary over time with disease progression and agonist exposure, which should be considered when interpreting our findings.

Our homogeneous population could reduce potential confounding factors related to genetic and ethnic diversity. However, further independent studies in other centers or populations are needed to validate our results, including prospective data collection with standardized clinical evaluation test during follow‐up, and exploring additional genetic candidates to improve clinical genetic predictive accuracy.

In summary, our study highlights the role of different clinical factors and also the DRD4 gene in the development of ICD in patients with PD. Specifically, we found evidence of the DRD4 7+ polymorphism and certain demographic and clinical factors as potential predictors of ICD consequences. This insight may pave the way out for developing personalized treatment strategies that target these risk factors, ultimately leading to improved quality of life for affected patients.

## Funding Information

This work was supported by the María de Maeztu program (grant #MDM‐2017‐0729) to the Parkinson's disease and Movement Disorders group of the Institut de Neurociències (Universitat de Barcelona), and Centro de Investigación Biomédica en Red de Enfermedades Neurodegenerativas (CIBERNED), 28031 Madrid, Madrid, Spain. Viviana Torres was a recipient of Programa Beca crédito Colfuturo, Bogotá, Colombia.

## Conflict of Interest

The authors have no conflict of interest to declare.

## Author Contributions

(1) Conception and design of the study: Conception (FV and ME); organization (VT, FV, ME, and RFS); execution (MF, VT, and ME); patient recruitment (YC, MJM, FV, VT, and AC). (2) Acquisition and analysis of data: Design (JR, ME, VT, JPM, and AG); execution (MF, VT, and JPM); interpretation: (JR, ME, FV, and VT); review and critique (all authors). (3) Drafting of the manuscript or figures: Writing of the first draft (VT, ME, JPM, and FV); draft editing (YC, MJM, and RFS); review and critique (all authors).

## Code Availability

All code used for this study is available upon reasonable request to the corresponding author.

## Supporting information


Appendix S1.

## Data Availability

All data that support the findings of this study are available upon reasonable request to the corresponding author.
