# Supplementary material for: DRD4 gene polymorphism and impulse control disorder induced by dopamine agonists in Parkinson's disease
Source: Ann Clin Transl Neurol. 2024 Jul 1;11(8):2222–9. doi: 10.1002/acn3.52111 (PMC11330210; doi:10.1002/acn3.52111)
Supplement: Supplementary file 1 — Appendix S1. [file ACN3-11-2222-s001.docx]

**Supplementary Methods: Clinical Data Collection Procedures**

We provide further details about essential aspects of our research methodology. Our goal is to offer a comprehensive understanding of the procedures we employed.

In our study, the screening for impulse control disorders was conducted during routine visits by neurologists who specialized in movement disorders and possessed extensive experience in this field. Given the retrospective nature of our study, our access was limited to the tools available through the review of medical records. These tools encompassed interviews focused on neuropsychiatric symptoms and specific screening tools, such as the one utilized in our study, to evaluate the potential presence of impulse control disorders. In our clinical practice, if a case of impulse control disorder is identified during these assessments, the patient is subsequently referred to a neuropsychiatrist for further evaluation and potential diagnosis. However, it is essential to note that the initial screening process is carried out by our specialized neurology team, which was the focus of our review.

**Participant origin:** Our study focused on collecting self-reported data on geographical family origins. The patients were asked about the city of origin of their maternal and paternal grandparents. We defined “Europeans” where there exist at least 3 grandfathers confirmed coming from a European place. By using this definition, we found out that 94.61% of the 241 selected patients were from Europe (mainly Spanish)

**Rapid Eye Movement Behavior Disorder (RBD) Screening:** The presence or absence of RBD was determined based on the RBD-Single-Question Screen *(RBD1Q)*as part of our routine patient consultations. The question was, "Have you ever felt like you act out your dreams during sleep, such as punching, gesturing, or simulating movements like running?".

**Psychiatric Comorbidity Assessment:**

The definition of psychiatric comorbidities was established by considering the presence or absence of factors that have been associated with a higher risk of developing impulse control disorders (ICD), such as depression, anxiety disorders, bipolar disorder, and other mood disorders. To assess these comorbidities, we thoroughly reviewed medical records, as it is a routine part of our clinical practice evaluation process.

**ICD Behavioral Grouping and Statistical Analysis Strategy:**

To simplify the analysis and enhance interpretability, we opted to combine the behaviors of buying and gambling within a singular category. Their shared financial implication aspects influenced this choice. This amalgamation facilitated a streamlined statistical analysis, yielding a more focused and easily interpretable set of variables for examination.

**Dopaminergic treatment**:

For the statistical analysis, the conversion of the dose of dopaminergic agonists to the equivalent levodopa dose was performed.

**Supplementary Methods: Genotyping Procedure**

For each DNA sample processed, Dopamine D4 receptor gene polymorphism (DRD4 VNTR) was amplified using the primers 5'-GCGACTACGTGG TCTACT CG-3' (forward) and 5'-AGGACCCTCATGGCC TTG -3' (reverse) in a VeritiTM 96-well Thermal Cycle (Applied Biosystems #4375786).

The PCR mix was constituted in a total volume of 25 μL and consisted of: 1 μL of each primer (30 pmol/ μL); 4 μL of 1.25 mM dNTPs; 0.1 μL of 3.5U/ μLTaq expand polymerase (ref: 11732641001; Expand High Fidelity PCR System), 2.5 μL of PCR buffer with MgCl2 (ref: 11759167001; Expand High Fidelity PCR System ); 2 μL of DMSO; 1 μL of DNA at 100 ng/ μL; and 13.4 μL ml of distilled water. The cycling conditions were 7 min 94°C followed by 35 cycles of 1 min 94°C, 1 min 62°C, 1 min 72°C, with a final 7 min step at 72°C. The PCR products were mixture with GelRed® Nucleic Acid Gel Stain in a 1:1 ratio, electrophorized on a 2% agarose gel with a 100 bp DNA ladder and then visualized under UV light. All genotypes were analyzed independently by two different scientists. Any discrepancies were resolved by repeating the corresponding genotyping process.

Out of a total of 241 processed DNA samples, 65 underwent repetition due to discrepancies or lack of amplification. Among these, 58 samples were repeated once, 6 samples were repeated twice, and 1 sample was repeated 3 times.

**Supplementary Methods: Statistical analysis**

The statistical analysis of clinical results was carried out with R (version 4.2.2, R Foundation for Statistical Computing, Vienna, Austria). All p-values were two-tailed with a significance threshold set at 0.05. Descriptive statistics were applied to give an overview of patients' clinical and genetic characteristics. Categorical variables were expressed as absolute numbers and percentages, whereas quantitative variables were treated as continuous variables and described as mean with standard deviation (SD) or median with interquartile range (IQR). Only complete-cases were considered for the analysis.

We stratify patients according to the three groups: non-ICBs, ICBs, or ICDs and tested categorical and quantitative variables using the Fisher exact test and Mann-Whitney U test, respectively. Variables considered as potential risk factors included sex, age of PD onset, related psychiatric diseases, LEDD, DA dose, dyskinesia, RBD, and DRD4 7 +. Since no significant differences in any variable were found between non-ICDs and ICBs groups, we combined them for subsequent analysis comparing with ICDs (Supplementary Table 1), in order to facilitate the interpretation of the results. The resulting group of combining non-ICB and ICBs groups is called non-ICD.

DRD4 locus was tested for conformity with Hardy-Weinberg equilibrium using a chi-square test. HardyWeinberg R package (V.1.7.5) was used to achieve this based on ^1^. Because we were primarily interested in the effect of the 7R allele or more for ICD, we combined alleles in two categories identified as allele “+7R” or “-7R“based on (PMID: 36635358). This yielded three genotypes: -7R/-7R, +7R/-7R and +7R/+7R. Hardy-Weinberg equilibrium was accepted for ICD group (p= 0.440) and for non- ICD group (p = 0.507) independently and also for the full studied cohort (p = 0.674).

We conducted a survival analysis considering the years from DA therapy initiation to the earliest signs of ICB-ICD onset or right-censoring data (end of treatment, follow-up, or death). The Kaplan-Meier method was applied to graphically describe the patients' evolution, and the curves were compared with the Log-Rank test. Univariate Cox regression analysis was performed to estimate risk of developing ICDs using a hazard ratio (HR) and their 95% confidence intervals (95%CI) with the potential risk factors. Additionally, multivariate Cox regression analysis was used to determine the effect of DRD4 VNTR polymorphism accounting for the influence of other variables, including sex, age at PD onset, previous neuropsychiatric diseases, RBD, dyskinesias, max daily LED of DA and LEDDs. The inclusion of variables in the multivariable Cox regression model was guided by significance in univariate analysis. The event's possible risk factors were evaluated by mean Hazard Ratio (HR), 95% Confidence Interval (95% CI), and p-value. The R packages survival (V. 3.5.5) ,survminer (V.0.4.9) and ggsurvfit (V. 0.3.0) were used to perform the survival analysis.

Multivariate logistic regression was used to estimate the risk of ICD development according to the analysed variables by means of estimation of odds ratio (OR) and their 95% CI. We performed a binomial logistic regression model with a backward stepwise approach for variable selection. Finally, we performed a Receiver Operating Characteristic Curve (ROC) using the scores from the logistic regression model to evaluate our model's classification capability. R packages used for this analysis are pROC (V 1.18.4) and ROCR (V 1.0-11).

**Supplementary Figure 1.**Flowchart Illustrating patient selection and analysis process for Parkinson’s disease patients treated with dopamine agonist.

**
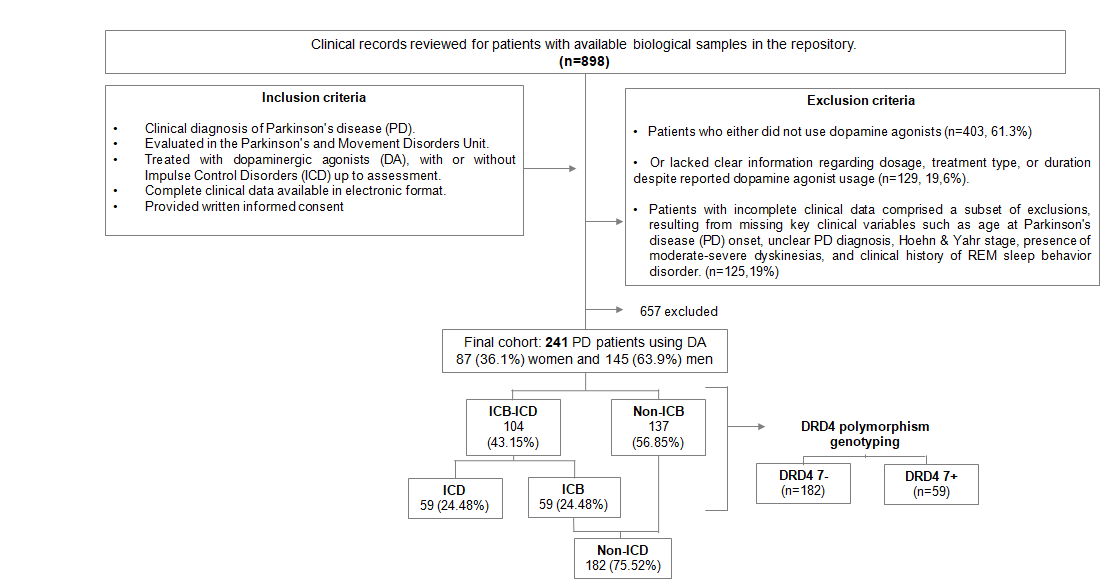
**

**Supplementary Table 1.**Fisher or Mann-Whitney test of non-ICBs versus ICBs.We analyzed categorical and quantitative variables using the Fisher exact test and Mann-Whitney U test, respectively. Variables considered as potential risk factors included sex, age of PD onset, related psychiatric diseases, LEDD, DA dose, dyskinesia, RBD, and DRD4 7 +.

| **Variables** | **p-value** |
| --- | --- |
| Sex | 0.4860 |
| Related psychiatric diseases | 0.7104 |
| DRD4 7+ | 0.6763 |
| Dyskinesias | 0.8336 |
| RBD | 0.0528 |
| Age of PD onset | 0.2211 |
| LEDD | 0.3003 |
| LED DA | 0.6197 |
|  |  |
|  |  |

**Supplementary Table 2.** Allele and genotype frequencies distribution for the DRD4 VNTR polymorphism in our cohort (N = 241).

|  | **All** | | **Women** | | | **Men** | |
| --- | --- | --- | --- | --- | --- | --- | --- |
|  | n | % | n | % | n | | % |
| **Allele** |  |  |  |  |  | |  |
| 2 | 54 | 11.2 | 19 | 10.92 | 35 | | 11.36 |
| 3 | 16 | 3.32 | 5 | 2.87 | 11 | | 3.57 |
| 4 | 342 | 70.96 | 124 | 71.26 | 218 | | 70.78 |
| 5 | 4 | 0.83 | - | - | 4 | | 1.30 |
| 6 | 1 | 0.21 | - | - | 1 | | 0.32 |
| 7 | 43 | 8.92 | 14 | 8.05 | 29 | | 9.42 |
| 8 | 18 | 3.73 | 9 | 5.17 | 9 | | 2.92 |
| 9 | 3 | 0.62 | 2 | 1.15 | 1 | | 0.33 |
| 10 | 1 | 0.21 | 1 | 0.58 | - | | - |
| Total | 482 | 100.00 | 174 | 100.00 | 308 | | 100.00 |
| **Genotype** |  |  |  |  |  |  |  |
| 4/4 | 133 | 55.19 | 48 | 55.17 | 85 | | 55.19 |
| 7/4 | 28 | 11.62 | 11 | 12.64 | 17 | | 11.04 |
| 4/2 | 20 | 8.30 | 7 | 8.04 | 13 | | 8.44 |
| 2/2 | 12 | 4.99 | 5 | 5.75 | 7 | | 4.55 |
| 8/4 | 12 | 4.99 | 6 | 6.90 | 6 | | 3.90 |
| 4/3 | 12 | 4.99 | 4 | 4.60 | 8 | | 5.19 |
| 7/2 | 8 | 3.32 | 2 | 2.30 | 6 | | 3.90 |
| 8/8 | 2 | 0.83 | 1 | 1.15 | 1 | | 0.65 |
| 7/3 | 2 | 0.83 | 1 | 1.15 | 1 | | 0.65 |
| 7/7 | 2 | 0.83 | - | - | 2 | | 1.30 |
| 5/4 | 2 | 0.83 | - | - | 2 | | 1.30 |
| 9/4 | 1 | 0.41 | - | - | 1 | | 0.65 |
| 7/5 | 1 | 0.41 | - | - | 1 | | 0.65 |
| 9/8 | 1 | 0.41 | 1 | 1.15 | - | | - |
| 8/2 | 1 | 0.41 | - | - | 1 | | 0.65 |
| 10/9 | 1 | 0.41 | 1 | 1.15 | - | | - |
| 3/3 | 1 | 0.41 | - | - | 1 | | 0.65 |
| 6/4 | 1 | 0.41 | - | - | 1 | | 0.65 |
| 5/2 | 1 | 0.41 | - | - | 1 | | 0.65 |
| 7- | 182 | 75.52 | 64 | 73.56 | 118 | | 76.62 |
| 7+ | 59 | 24.48 | 23 | 26.44 | 36 | | 23.38 |
| Total | 241 | 100.00 | 87 | 100.00 | 154 | | 100.00 |
|  |  |  |  |  |  | |  |

**Supplementary Table 3.** Summary of univariate and multivariate Cox regression model of overall ICD survival duration. Factors with or without statistical significance are shown in the univariate Cox regression. Only significant variables were introduced into the multivariate model.

|  |  | | | **Univariate Cox regression** | | | | | **Multivariate Cox regression** | | | | |
| --- | --- | --- | --- | --- | --- | --- | --- | --- | --- | --- | --- | --- | --- |
|  | |  | HR | 95% CI | | p-value | | | HR | 95% CI | | | p-value |
| Sex | | Female | Ref. |  | |  | | | Ref. |  | | |  |
|  | | Male | 2.38 | 1.18-4.41 | | **0.006 *** | | | 2.88 | 1.53-5.41 | | | **<0.001 *** |
| Age of PD onset | | Years | 0.96 | 0.94-0.98 | | **<0.001 *** | | | 0.97 | 0.94-0.99 | | | **0.007 *** |
| Related psychiatric diseases | | No | Ref. |  | |  | | | Ref. |  | | |  |
|  | | Yes | 2.60 | 1.11-6.09 | | **0.028 *** | | | 2.84 | 1.18-6.84 | | | **0.020 *** |
| Dyskinesias | | No | Ref. |  | |  | | | Ref. |  | | |  |
|  | | Yes | 2.27 | 1.35-3.80 | | **0.002 *** | | | 2.00 | 1.18-3.39 | | | **0.010 *** |
| DRD4 7+ | | No | Ref. |  | |  | | | Ref. |  | | |  |
|  | | Yes | 2.00 | 1.18-3.39 | | **0.010 *** | | | 1.95 | 1.14-3.34 | | | **0.015 *** |
| RBD | | No | Ref. |  | |  | | |  |  | | |  |
|  | | Yes | 1.55 | 0.93-2.59 | | 0.092 | | |  |  | | |  |
| LEDD | | g | 0.98 | 0.91-1.06 | | 0.654 | | |  |  | | |  |
| LED DA | | g | 1.13 | 0.94-1.35 | | 0.192 | | |  |  | | |  |
|  | |  | |  |  | |  |  | | |  |  | |
| **CI**: Confidence interval, **RBD**: REM behavior disorder, **DA**: Dopamine agonist, **LEDD**: Levodopa equivalent daily dosage  Bold values indicate statistically significant differences (p-value* < 0.05) | | | | | | | | | | | | | |

**Additional References:**

1. Graffelman, J. (2015). Exploring Diallelic Genetic Markers: The HardyWeinberg Package. Journal of Statistical Software, 64(3), 1–23. <https://doi.org/10.18637/jss.v064.i03>
2. Clochard, G. J., Mbengue, A., Mettling, C., Diouf, B., Faurie, C., Sene, O., Chancerel, E., Guichoux, E., Hollard, G., Raymond, M., & Willinger, M. (2023). The effect of the 7R allele at the DRD4 locus on risk tolerance is independent of background risk in Senegalese fishermen. Scientific reports, 13(1), 622. https://doi.org/10.1038/s41598-022-27002-3
